# Laser-patterned metallic interconnections for all stretchable organic electrochemical transistors

**DOI:** 10.1038/s41598-018-26731-8

**Published:** 2018-05-31

**Authors:** Bastien Marchiori, Roger Delattre, Stuart Hannah, Sylvain Blayac, Marc Ramuz

**Affiliations:** Mines Saint-Etienne, Centre of Microelectronics in Provence, Department of Flexible Electronics, F-13541 Gardanne, France

## Abstract

We describe a process allowing the patterning of fully stretchable organic electrochemical transistors (OECTs). The device consists of an active stretchable area connected with stretchable metallic interconnections. The current literature does not provide a complete, simple and accurate process using the standard thin film microelectronic techniques allowing the creation of such sensors. An innovative patterning process based on the combination of laser ablation and thermal release tape ensures the fabrication of highly stretchable metallic lines – encapsulated in polydimethylsiloxane – from conventional aluminium tape. State-of-the-art stretchability up to 70% combined with ultra-low mOhms resistance is demonstrated. We present a photolithographic process to pattern the organic active area onto stretchable substrate. Finally the formulation of poly(3,4-ethylenedioxythiophene):poly(styrenesulfonate) is tuned to achieve an OECT with a maximum stretchability of 38% while maintaining transconductance up to 0.35 mS and channel current as high as 0.2 mA.

## Introduction

Conventional electronic components are mostly based on the combination of silicon-based semiconductors and metallic materials. They are predominantly rigid and stiff, rendering them inflexible and not stretchable. On the other hand, flexible electronics systems based on for example, polyethylene terephthalate (PET), polyimide (PI) and polycarbonate (PC) substrates, can undergo some deformation and conform to bent objects^1^, but require a particular arrangement to conform to 3-dimensional surfaces^2^. Recently, stretchable electronics has been emerging as an extension of flexible electronics^3^. The stretchability aspect allows electronic devices to conform easily to complex structures and can for example, emulate the mechanical properties of the skin. Therefore, stretchable electronic devices are particularly interesting for the development of various sensing capabilities such as temperature, touch, electrocardiogram and motion detection in the framework of electronic skin applications^4,5^ or bioelectronics for sensing at the interface with the living^6^.

A prerequisite to creating stretchable electronic components is to develop materials that can withstand large mechanical constraints. To help achieve this, polymers with elastomeric properties are often chosen as substrates^7^. For bioelectronics applications, polydimethylsiloxane (PDMS) is widely used since it is a silicone-based polymer and is biocompatible, commercially available, low cost, solution processable with hyperelastic properties and is also stretchable to more than 100%^8^. The integration of stretchable metallic interconnections onto hyperelastic materials was recently the subject of intensive research^9^. The achievement of high quality metallic conductive lines on stretchable substrates relies on two main parameters: the mechanical properties of the material itself and the line geometry.

Concerning the material, researchers have developed elastic conductors made from elastic materials, filled with conductive nanoparticles. For example, Kim *et al*. have reached a conductivity of 10^3^ S cm^−1^ with Ag nanowires in polyurethane (PU), featuring a maximum strain of 100% and 50 S cm^−1^ after almost 500% strain^10^. Recent work has demonstrated a Ag flakes based ink on PDMS with a conductivity of 182 S cm^−1^ at 215% strain^11^. Composite polymeric materials have the advantage of being highly stretchable, but offer lower conductivity than bulk metals (6 × 10^5^ S cm^−1^ for Copper and Silver).

Another field of investigation is the evaporation of gold on PDMS, used for its biocompatible properties. Lacour *et al*. showed the first reported stretchability of gold evaporated lines on PDMS, whereby the resistance increased by a factor of 8 after 8% strain was applied^12^. In a further step, they demonstrated 3 mm-wide gold lines showing no increase in resistance up to 100% stretchability by using a substrate prestrain method during evaporation^13^. This prestrain induces wrinkles on the surface of the materials when released, improving the stretchability of the material^14,15^. Recent work has introduced the use of Gallium as a liquid metal at the interface of gold to make it stretchable without requiring prestrain, displaying stable resistance after multiple cycles at 50% strain^16^.

A specific geometry is needed to provide stretchability when using rigid conductive materials. Gray *et al*. showed that it is possible to create in-plane electroplated gold stretchable interconnections via photolithography by using a horseshoe shape that can withstand a strain up to 50%^17^. Moreover, optimisation of the geometry has led to embedded copper lines in PDMS with a stretchability up to 100% and a change in resistance of less than 5%^18^. Out of plane 3D metallic interconnections were also investigated featuring a stretching capability up to 140%^19,20^.

An alternative to photolithography has been found with the design of serpentines made out of flexible polymers as a substrate for the deposition of metallic thin-films^21^. The Vanfleteren group has already demonstrated the use of laser patterning a flexible circuit board embedded within PDMS for stretchable applications^22^, wherein initially, a metal layer is deposited on a flexible polymer (PI/PET). Next, a temporary adhesive is used to fix and flatten the substrate. Finally, a laser beam is used to selectively pattern the interconnection. Their results exhibit between 80% and 100% stretchability. A study of the optimisation of the line parameters (geometry, materials) has been partially investigated^23^. Using a technique based on electronic cutting, Yang *et al*. have demonstrated indium tin oxide on PET serpentines displaying a stretchability up to 100%. Despite the high stretchability of the film, the resistance increases rapidly due to the appearance of cracks in the thin-film^24^.

To maximise the potential stretchability of the serpentines, the material has to be weakly bonded to the substrate^21,24^. Due to the mechanism of deformation, the serpentine has to be able to deform out of the plane of the substrate^25,26^. Thus, this method is not applicable in the case of the physical deposition of thin-films with no self-standing capability.

In this study, we have developed a process to fabricate highly conductive stretchable interconnections featuring a very stable resistance under the influence of stretch to fabricate a fully stretchable organic electrochemical transistor (OECT). The transistor is based on a poly(3,4-ethylenedioxythiophene): poly(styrenesulfonate) (PEDOT:PSS) conducting polymer and has wide ranging applications within bioelectronics. From glucose sensing^27^ to cell monitoring^28–30^, the OECT has been largely studied for its ability to transduce ionic current to electrical current^31–34^. Such devices are usually fabricated on non-stretchable materials^35^. However, the OECT has significant potential to act as an interface with biological systems^36^, therefore, making it stretchable/conformable provides a superior biological/electronic interface and improves the recording signal quality, in particular for *in vivo* recording.

To produce high-performance transistors, the most effective process is based on photolithography which provides high resolution patterning. However, the use of photolithography directly on PDMS has shown limitations^37^. Some alternative methods have been developed to pattern active materials on elastomers with transfer patterning^38^, screen printing^39^, inkjet printing^40^ or stamping^41^, but are limited in resolution and difficult to implement for multi-layer alignment.

The first demonstration of a highly stretchable, albeit out of plane OECT was shown five years ago^42^. Despite its high stretchability up to 270%, the non-coplanar structure of the OECT makes biointerface applications a challenge. A fabrication process for an in-plane stretchable device has recently been developed based on a combination of parylene transfer and photolithography by using an orthogonal photoresist on a hydrogel, leading to an OECT which is stretchable up to 30% without cracking as a result of a prestrain step before fabrication^43^. However for this device, only a modest transconductance of 0.6 mS was achieved. Moreover, OECTs are stretchable only up to the initial prestrain load. Past this value, the mechanical integrity of the device is compromised, leading to the manifestation of cracks.

In this paper, we describe a novel approach to fabricate a fully stretchable OECT, which includes the development of a new fabrication process for stretchable conductive interconnections and the optimisation of the PEDOT:PSS active material. To ensure compatibility with conventional microfabrication processes, we avoid the use of a prestrain step that is commonly used to improve device stretchability^44^. We overcame the challenge of photolithography on PDMS by keeping the non-stretchable layer of Pa-C during the fabrication process that serves to pattern the active area. We report superior combined electrical and mechanical performance of the stretchable OECT compared to previously published work.

## Results and Discussion

### Fabrication of the stretchable interconnections

**Figure 1:**
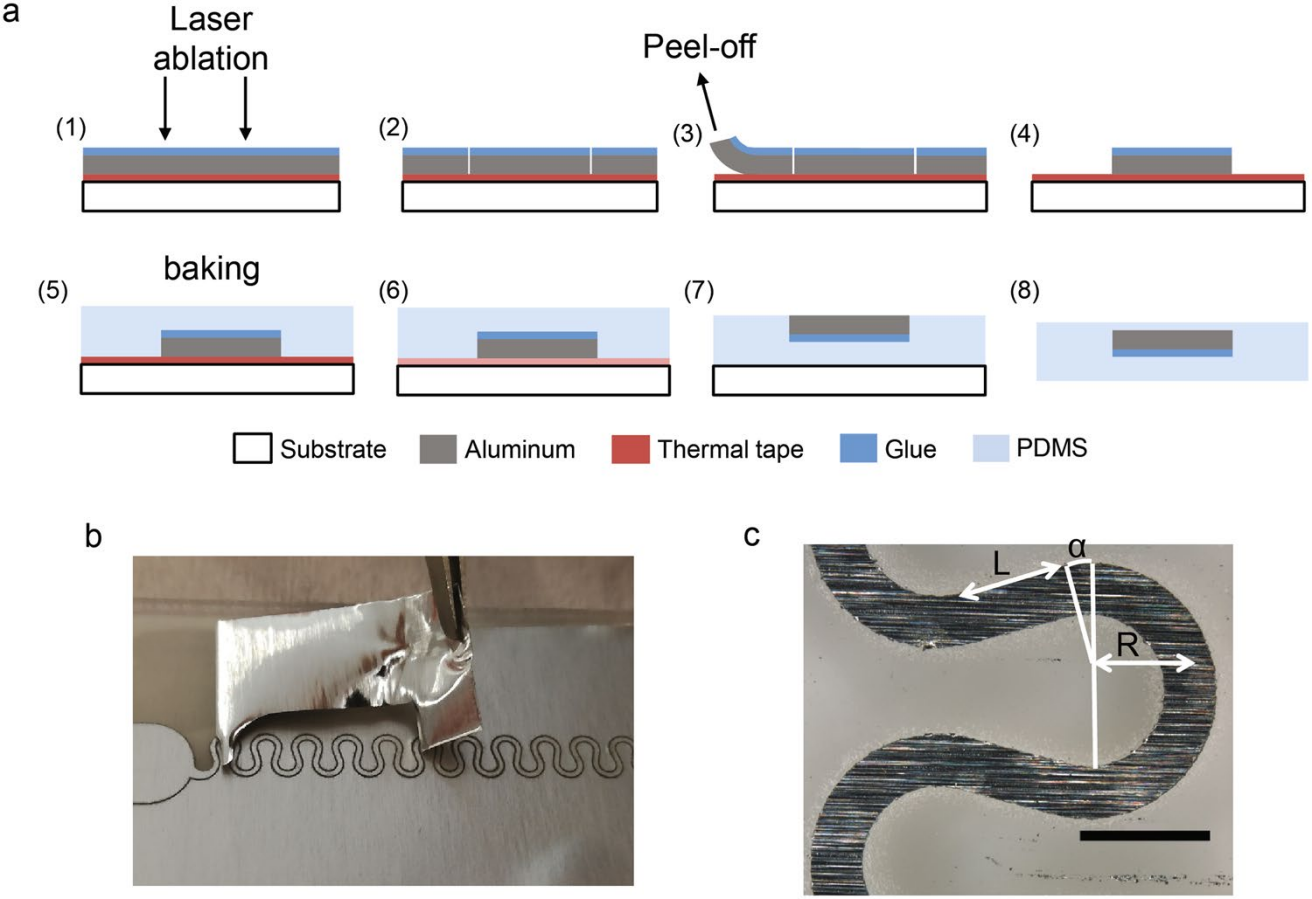
Fabrication and characterisation of stretchable interconnections. (**a**) Schematic of the preparation process of the metallic line based on the laser cutting of metallic foil attached on a thermal release tape. Once patterned and peeled off, the line is encapsulated in PDMS. The final device consists of aluminium tape sandwiched within PDMS layers, which offer a high degree of stretchability. (**b**) Illustration of the peel-off process. The unwanted regions of metal are either manually removed or removed by the laser to have a process compatible with conventional thin-film microelectronics fabrication. (**c**) The sweep on the different parameters is illustrated: the angle α, the length of the connections L between two arcs of the circles and R, the radius of the arc of a circle. The aluminium thickness is 50 µm and the width of the lines is 400 µm. The scale bar represents 1 mm.

We have developed an innovative process for the fabrication of stretchable interconnections encapsulated in PDMS. It is based on the laser patterning of aluminium tape as shown in Fig. 1a. First, a metallic 50 µm-thick aluminium tape or sheet is laminated on a glass slide using a thermal release double-sided Nitto RevAlpha® tape sandwiched in between. This tape allows the aluminium to be perfectly planar on the surface during the fabrication process. The metallic interconnections were patterned using direct laser ablation. A video of the laser processing procedure is available in supplementary information (Fig. S1). This method consists of the localised ablation of the metal layer to outline the circuit. The aluminium tape was cut with the laser through the glue (1–2) and subsequently, the unwanted metallic regions were peeled off (3–4) from the thermal release tape substrate as illustrated in Fig. 1b. As an alternative method, it was found to be possible to completely remove the superfluous metal by heating the tape through the aluminium with the laser. Next, after pouring and curing the PDMS layer (5), the tape was thermally released (6), and the resulting device was laminated upside down on a glass substrate (7) to completely seal it into an encapsulation layer of PDMS with a final spin coating process (8). We ensured this layer was thick enough to prevent the line from detaching from the substrate^25^. The resulting thickness of the encapsulated line into PDMS was 700 µm. Following this procedure, we were able to pattern high-resolution stretchable bulk metal interconnections with outstanding repeatability. The resolution of the line is limited by both the dimension of the laser beam (25 µm) and the thickness of the metallic foil. Higher resolution can be achieved using advanced laser cutting techniques^45^.

Compared to other examples of patterned stretchable metallic connections, the advantages of the aforementioned process are its simplicity since the process does not use a mask, the compatibility with microfabrication at an industrial scale-production and its versatility. We also successfully tested this process with other metallic foils, such as copper, titanium, etc… We were able to achieve superior lateral resolution than previous works on laser patterning^23^ since we only use and cut a layer of metal instead of a combination of PI/metal or PET/metal. Indeed, the ablation of the polymer with the laser provokes burning on the sides, reducing the final resolution. The use of the thermal release tape in step 5 provides perfect control of the planeness for the fabrication of the device, which is essential for the deposition of thin active layers that are only 300 nm thick and when using relatively thicker metallic sheets of 50 µm.

### Characterisation of the stretchable interconnections

To ensure stretchability of the metallic lines, we use a horseshoe wire design for the stretchable interconnections which have been investigated previously^9,18,46^. Upon application of strain, the line acts like a spring, whereby it unfolds gradually.

To maximise the extension applied to the system, we concentrated on optimising the geometrical parameters of the horseshoe presented in Fig. 1c: the angle of the arc α called angle of routing, the radius of the arc R and the length of the connection between two arcs L. While varying one of the parameters, the others were fixed to either α = 15°, L = 800 µm, or R = 800 µm. From this variation, it is possible to calculate the maximum theoretical stretchability ε that can undergo a free serpentine from a coplanar stretch. This strain is defined as the maximum percentage of its elongation before becoming completely straight. It is expressed by equation (1); the normalised ratio of the line stretched out to the length of the equivalent straight line^9^:

1 $$\varepsilon =\frac{2(\alpha +\frac{\pi }{2})+\frac{L}{R}}{2\,\sin (\alpha +\frac{\pi }{2})+\frac{L}{R}\,\cos (\alpha +\frac{\pi }{2})}-1$$

**Figure 2:**
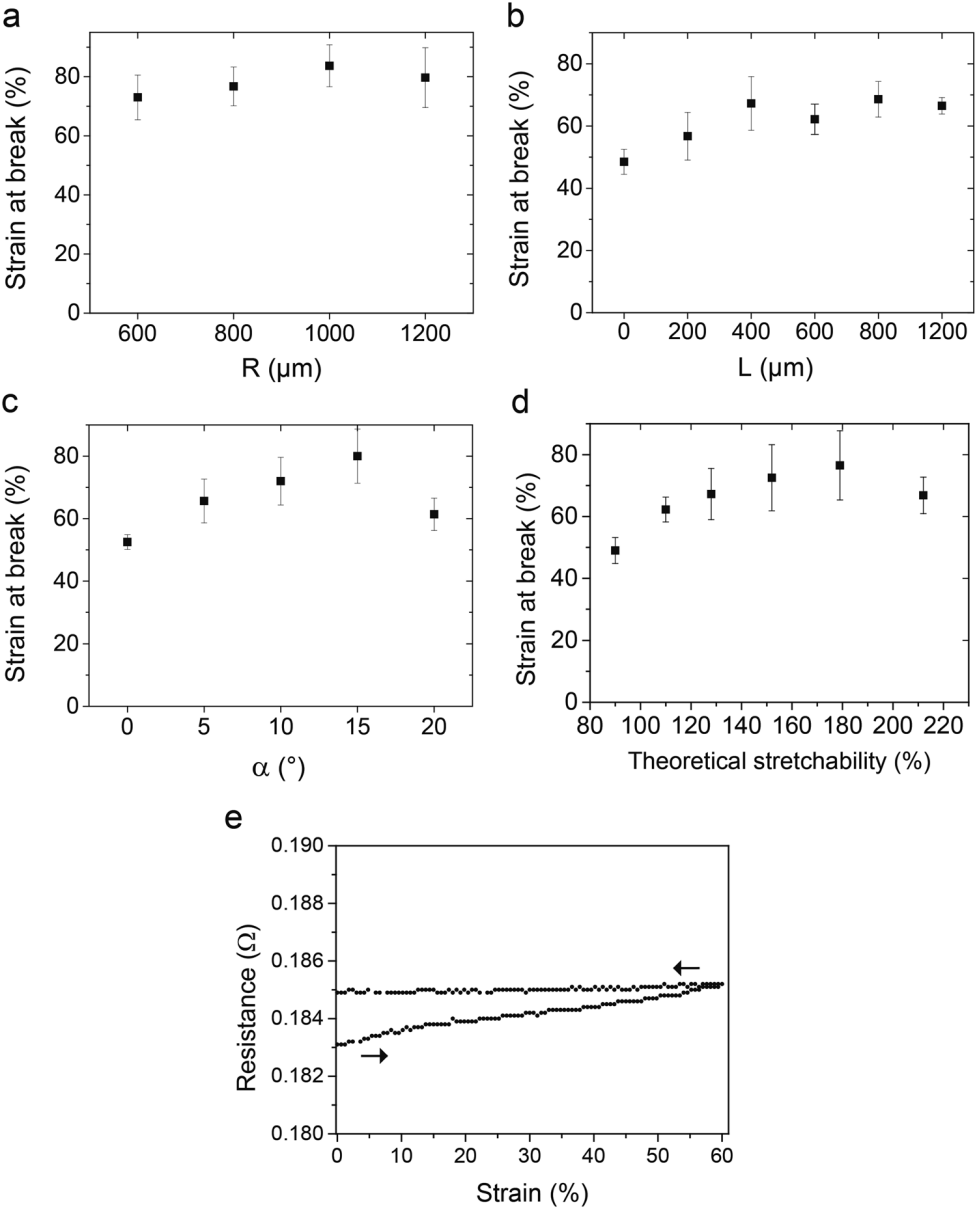
Optimisation of the stretchability of the interconnections. (**a**–**c**) Experimental strain at break of the line for each parameter over 10 samples. The strain is applied at a speed of 100 µm/s and a measurement of the electrical resistance is taken at every 0.5% strain with a Keithley® using a four-terminal measurement setup. (**d**) Compilation of the previous results representing the strain at break as a function of the calculated theoretical stretchability which is equivalent to the maximum strain the line can undergo before becoming straight. It is possible to design stretchable interconnections with an average point of rupture of 70%. (**e**) Example of electrical/mechanical characterisation of a line with the resistance as a function of the strain up to 60% for a line with width = 400 µm, α = 15°, L = 800 µm, R = 800 µm, metal thickness = 50 µm for a total length of the serpentines of approximately 95 mm. Distance between the jaws is 40 mm. The strain is applied up to 60% and then goes back to 0%. There is an increase of 1% in the resistance while stretching due to small strain in the metal but afterwards, the resistance stays constant during relaxation from 60 to 0%.

The mechanical study performed on the lines shows a difference between the theoretical and experimental behaviour as presented in Fig. 2. The encapsulation prevents the line from deforming out of the plane, leading to an early break^25^. For each parameter and over ten separate samples, the impact of the shape of the constrained horseshoes on the maximal point of rupture was studied, which was then correlated to the theoretical stretchability value.

Referring to Fig. 2a, the changes in the radius R have a limited impact on the strain at break of the line. The stretchability of the line with R = 1000 µm is the highest, nevertheless showing a moderate impact of this parameter in this range of data. Concerning the length L, Fig. 2b shows the effect on the mechanical properties for L ≤ 1200 µm. The strain at break is improving (increasing) as a function of increasing L up to 400 µm. However, after this value, the point of rupture is becoming more constant. For the last parameter α shown in Fig. 2c, we also observe an increase of the mechanical properties, up to 15°. However, for α = 20°, the line is breaking earlier at a lower strain at break value. While the total length of the line is longer, we would expect the interconnection to be more stretchable with such an angle; but it is the opposite. This effect has already been reported before by Hocheng *et al*.^9^. The hypothesis is that before α = 15°, the line is breaking due to the tension in the metal; and after α = 20°, compression of the PDMS substrates on the sides is causing the rupture of the lines.

We then calculated the theoretical stretchability (equation 1) associated with the data shown in the graphs of Fig. 2a,b,c and compared them to the experimental strain at break as seen in Fig. 2d. The geometry is improving the mechanical performance for equivalent theoretical stretchability up to 140%. After this value, the impact is negligible, except for the last data point at 210% which has a lower resistance to strain due to the same reasons explained previously for α = 20°.

To achieve maximum stretchability with the interconnections, we identified the key parameters of the geometry for the horseshoes. The tuning of the angle of routing α has the highest impact on the break of the lines, with an optimum angle of 15°. We have also seen that L must be greater than 400 µm to achieve the best stretchability. Finally, even if the choice of the radius of the horseshoe will have a reduced impact on the mechanical performance of the line, we can choose R = 1000 µm to maximise them. By linking these parameters to the resulting theoretical stretchability, we have shown that we can estimate the strain at break of the interconnections. There is a range between 140% and 180% of theoretical stretchability in which the rupture of the line stays similar when tuning the geometry. These interconnections have a measured average stretchability of 70%. This value is roughly half of theoretical stretchability, which can be mainly attributed to the encapsulation of the line. During the strain, the line tends to deform out of the plane, which the encapsulation layer does not allow. This results in an increase in the stress in the line and explains the earlier break^25^.

Figure 2e illustrates the electrical behaviour of a stretchable line during a uniaxial tensile test. During the stretch, the resistance increases by only 1% from 183 mΩ to 185 mΩ up to 60% elongation. Then, the resistance stays stable during the relaxation period of the line. The resistance is increasing due to the development of irreversible deformations in the line such as cracks^25^. Comparing this behaviour to the literature^13,18^, the resistance is 10 to 100 times lower and is stable while stretching because we pattern thick, pure metallic sheets. It is also possible to get high stretchability using composite polymers, however the conductivity is around 1000 times smaller and it decreases under stretching^10,11^. The ultra-low resistance of our state-of-the-art interconnections allows their use for low voltage sensing which is particularly crucial for enhancing OECT performance and for biosensing applications.

### Process and optimisation of the OECT

**Figure 3:**
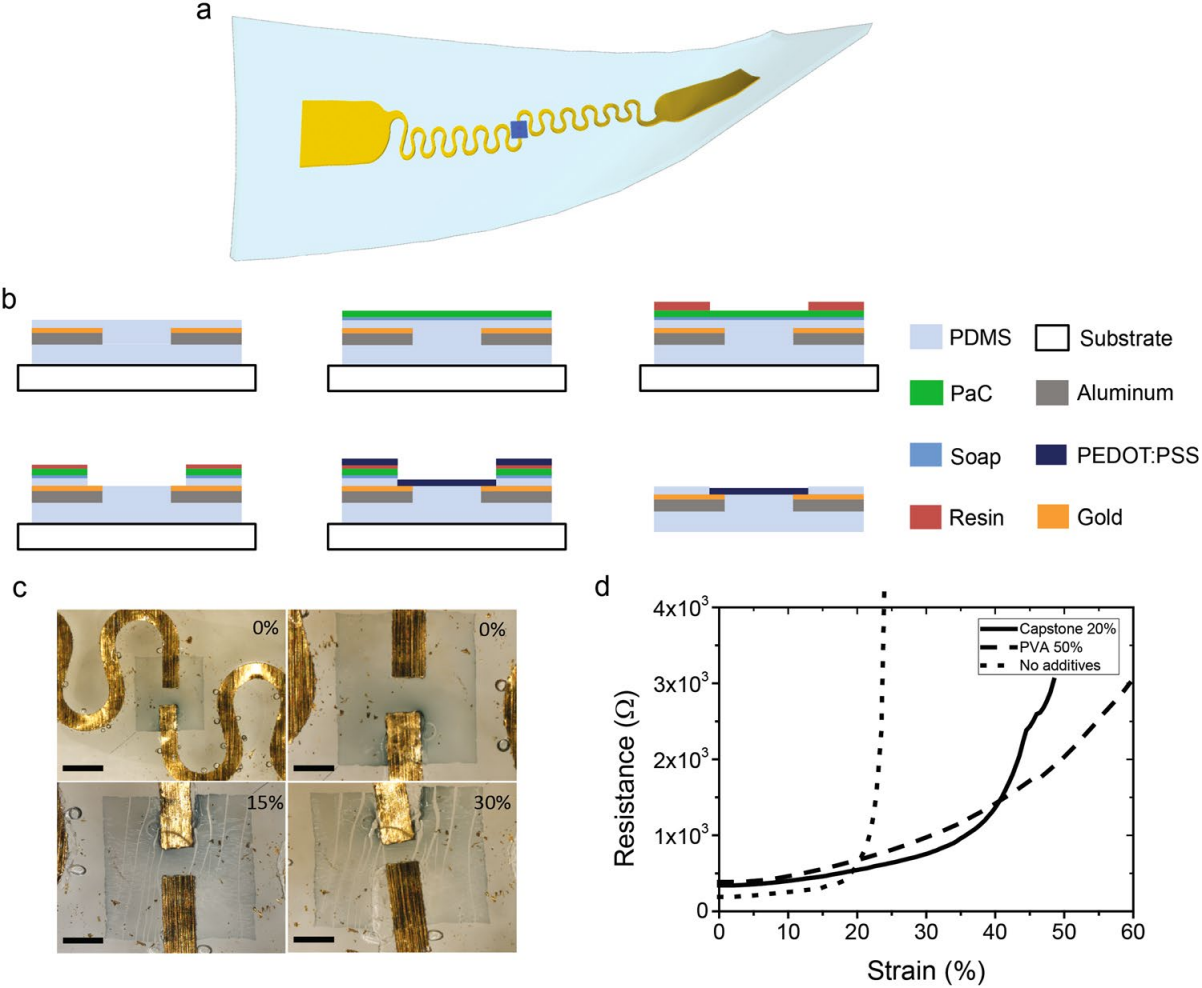
Process detailing the fabrication of a stretchable OECT. (**a**) Resulting device modelled in 3D (**b**) Schematic depicting the fabrication steps. The encapsulated lines are covered by parylene, then, the opening of the channel and contacts on pads are done by photolithography and reactive ion etching of parylene and the PDMS encapsulation layer. The PEDOT:PSS formulation is then spin-coated and the sacrificial parylene layer is removed to pattern the active area. The resulting device is a fully stretchable OECT including metallic lines, PEDOT:PSS and PDMS. (**c**) Picture of the channel at the end of the process for different strains. Scale bar is 1 mm for the 0% strain picture and 500 µm for the others. It is evident that some cracks are appearing in the active area, but the channel zone is not affected. (**d**) Optimisation of the formulation of the OECT: Resistance of a square of 17 mm by 25 mm of PEDOT:PSS layer on PDMS as a function of the strain. The addition of Capstone as a fluorosurfactant increases the mechanical properties of the PEDOT:PSS. Furthermore, the addition of PVA also enhances the mechanical properties, but ultimately appears to delaminate from the substrate whilst under stretch.

We developed a process based on the most common way of making an OECT, namely the parylene-C (Pa-C) lift-off technique reported in the literature^35,47^. A 3D design of the resulting encapsulated device is displayed in Fig. 3a. A schematic of the fabrication process is depicted in Fig. 3b, where Pa-C was used as a sacrificial layer and support for the photolithography process. Rather than using Pa-C as a second layer for the insulator which is not stretchable, a thin (11 µm) PDMS spin-coated layer was used by mixing PDMS with hexane (1:1 in mass). The encapsulated line made of aluminium covered with 100 nm of gold was coated with a soap solution acting as an anti-adhesive. Next, the Pa-C layer was deposited and patterned by photolithography and plasma etching respectively with an opening at the contact pads and in the channel. The PDMS encapsulation layer was also etched with a mixture of O_2_ and SF_6_^48,49^ to open the connections on the pads and on the channel. The PEDOT:PSS mixture was spin coated, and the sacrificial Pa-C layer was subsequently peeled-off from the PDMS device.

The metallic interconnection was cut in the middle with the laser to pattern the channel. As displayed in Fig. 3a, the channel was designed perpendicular to the strain direction to minimise the extension of its length during the strain. From Fig. 3c, we observe a diminution of the channel length due to the compression of PDMS on the side. Some cracks are appearing, but only out of the channel, keeping its integrity and so, its ability to successfully conduct current.

The formulation of PEDOT:PSS mixture for the OECT channel is based on work done by Sessolo *et al*. with the addition of ethylene glycol, 4-dodecylbenzenesulfonic acid (DBSA) and (3-Glycidyloxypropyl)trimethoxysilane (GOPS) to improve its conductivity adhesion^35^. This formulation has been developed for rigid/flexible OECTs, but not for a stretchable one. With this in mind, we investigated two main chemicals: a fluorosurfactant (capstone®) and polyvinyl alcohol (PVA) since these two compounds are known to increase the stretchability of PEDOT:PSS films^50–52^.

The solution was spin-coated on a PDMS sample after treatment with O_2_ plasma. The resistance of a layer of PEDOT:PSS mixture on PDMS was evaluated as a function of the strain for different amounts of additives. Graphs of the different experiments for the PEDOT:PSS mixture optimisation are available in the supplementary information (Fig. S2). As shown in Fig. 3d, without any additives, the formulation described previously has a stable resistance until 20% strain. After this value, the resistance increases rapidly, and the layer resistance is too high to provide good performance as an OECT. The impact of PVA on the stretchability of the film was investigated with solutions from 0 wt% to 70 wt% without the addition of GOPS. Its addition resulted in an increase in resistance and provided no improvement in the mechanical properties. With regards to the literature, it appears that the addition of PVA does not affect the mechanical properties before 40 wt% of PVA^52^. The best mechanical properties are found to be with 50 wt% PVA, but it appeared that the PEDOT:PSS mixture layer was delaminating from the PDMS substrate while stretching. This is most likely due to the absence of GOPS which acts as a cross-linker agent, helping with the adhesion of the film^53^. The impact of the addition of Capstone between 0 vol% and 40 vol% is available in the supplementary information (Fig. S2). The best ratio of Capstone is 20 vol%, showing high conductivity and an increase of the resistance at around 35% strain. A study of the polymer at the molecular level is needed to thoroughly understand the impact of the different additives on the mechanical and electrical properties.

Since the PVA based solution became delaminated under stretching, we selected 20%vol Capstone as an additive for improving the mechanical properties of the active area. It resulted in an active area with high stretchability and a conductivity of 150S cm^−1^; close to the highest but non stretchable conductivity of a PEDOT:PSS mixture (900S cm^−1^)^54^.

### Characterisation and performance of the OECT

With these optimised metallic interconnections and PEDOT:PSS formulation designed for improved stretchability, we fabricated a high performance OECT. A picture of a device under test is available in the supplementary information (Fig. S3).

**Figure 4:**
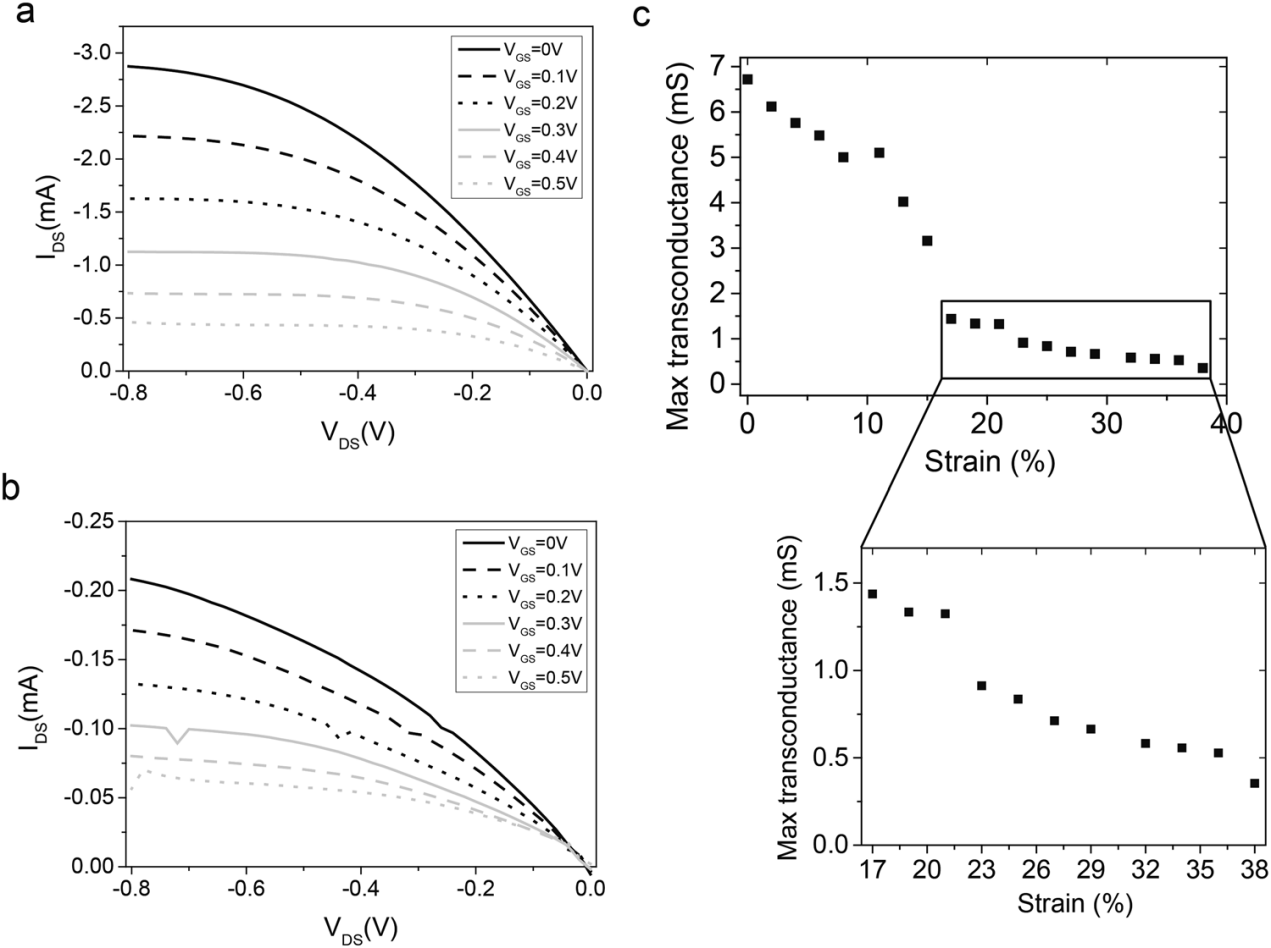
Characterisation of an OECT with a channel length of 250 µm and a thickness of 300 nm with an external Ag/AgCl gate. (**a**,**b**) Output curve of the device with V_GS_ varying from 0 V to 0.5 V. (**a**) At 0% strain and (**b**) 38% strain. The device clearly displays transistor behaviour showing high current even at 38%. Nevertheless, the current has decreased by a factor 7. (**c**) The maximum transconductance as a function of the strain extracted from the IV curves of the best performing device with an initial transconductance around 6.5 mS at 0% strain. There is a noticeable reduction in the performance after 11% strain. Focussing on the zoomed-in part in the graph of (**c**), for strains between 17% and 38%, the transconductance is diminishing slowly from 1.5 mS to 0.35 mS. After this value, it is not possible to record a proper output curve and therefore extract the transconductance.

In Fig. 4a, we present a typical output characteristic from an OECT at 0% strain, with a drain-to-source voltage (V_DS_) swept from 0 to −0.8 V, for a gate-to-source voltage (V_GS_) ranging in magnitude from 0 to 0.5 V in 0.1 V increments. The length of the OECT channel is 250 µm, the contact lines are 400 µm wide and the total device thickness is 300 µm. For the best transistor, we obtained a maximum drain current magnitude of around 2.8 mA. Good transistor behaviour, with a current magnitude of 0.2 mA, was still observed until 38% strain (Fig. 4b). More output curves are available in the supplementary information (Fig. S4) for several different strain values. To characterise the performance of an OECT, the transconductance (gm = ΔI_DS_/ΔV_GS_) is generally used as a useful figure of merit and corresponds to the ability of the OECT to be de-doped under gate bias. A high gm ensures a high OECT gain, and thus a high sensitivity when used as a sensor. The maximum transconductance associated with the output curves for each strain applied is displayed in Fig. 4c. On this graph, we chose to display only the maximum transconductance, which in our case, corresponds to the gain between a gate voltage magnitude of 0 V and 0.05 V (Fig. S5). We observe a slight decrease down to 5 mS at an elongation of 11%, after which, there is a significant drop between 11% and 17% where the transconductance falls to 1.5 mS, and finally, in Fig. 4d, we can observe a slow decrease to 0.35 mS until 38%. Further examples and more details of the characterisation of other devices are available in the supplementary information (Fig. S6).

The maximum transconductance of 6.5 mS is comparable to rigid based OECTs fabricated on glass^34^. The drop in OECT performance after 11% strain has been observed previously. For strain higher than 10%, cracks start to appear in the PEDOT:PSS films and so, it would be fair to assume that the elastic limit has been reached, thereby entering the plastic region^51^. This deterioration in performance was not seen in Fig. 3d where the PEDOT:PSS mixture film resistance was fairly stable and lower than 1000 ohms until 40% strain. As a result, the drastic reduction of transconductance can be attributed to the interface between the PEDOT:PSS layer and the metal. Since these two materials have different mechanical properties (Young’s Modulus), there is an accumulation of stress at this interface which is detrimental to the overall performance of the OECT.

## Conclusion

We have developed a process to pattern a stretchable electrochemical transistor. The innovative process of laser cutting bare metallic foils produces conductive interconnections embedded in PDMS, featuring a maximum stretchability around 70% without change in the resistance. The low resistivity of these lines is particularly suitable for biosensors, where the applied voltage has to be kept to a minimum to avoid water hydrolysis. To the best of our knowledge, this is the first demonstration of bulk metal interconnections embedded in PDMS showing combined high stretchability and high electrical performance. By taking advantage of these interconnections, we have developed a process for the patterning of an OECT based on the lift-off of a sacrificial Parylene-C layer by replacing non-stretchable materials with PDMS, patterned by photolithography and dry etching. This process is compatible with standard microfabrication techniques; it avoids any prestrain step and contains only stretchable materials. The resulting device displays very high performance until 11% strain, with good transconductance and output characteristics until 38%. This work provides a significant step towards the implantation of sensors onto living organs for continuous recording. Beyond its high conformability, this device is also highly compatible with microfluidics which is a key step towards the production of accurate *in-vitro* models.

## Methods

### Fabrication of interconnections

Laser cutting was performed using LPKF Protolaser S equipment. The radiation used was 1064 nm with a frequency of 75 MHz and a power of 10 W. The diameter of the laser beam was 25 µm. The aluminium tape was laminated on a glass slide (7.62 cm × 2.54 cm) using a thermal release double-sided Nitto 90 °C RevAlpha tape sandwiched in between. The first layer of PDMS was spin coated at 300 rpm to achieve a thickness of 350 µm and was then cured at 70 °C for at least 3 hours. Afterwards, the RevAlpha tape was released in an oven at 100 °C for 15 minutes. After lamination of the first layer of PDMS, the spin coating of a 100 µm encapsulation layer was carried out at 1200 rpm.

### PEDOT:PSS formulation

The conducting polymer formulation consisted of PEDOT:PSS (Heraeus, Clevios PH 1000), with ethylene glycol (Sigma–Aldrich, 0.25 mL for 1 mL PEDOT:PSS solution), 4,dodecylbenzenesulfonic acid (DBSA) (0.5 μL mL^−1^), when specified, GOPS (10 mg mL^−1^), Capstone® FS-30 or PVA (Mw = 13000–23000 g mol^−1^, mother solution at 10%wt in water). The resulting dispersion was sonicated for 30 minutes and then spin-coated at 1500 rpm for 30 seconds. To optimise the formulation, the solution was spin-coated on a 3.81 mm by 2.54 mm PDMS sample after a mild plasma treatment at 25 W without oxygen using a PE-100 plasma system.

### OECT Fabrication

After fabrication of the interconnections, a layer of 3% Micro 90 soap was spin coated at 1500 rpm, followed by the deposition of a layer of Parylene-C using a SCS Labcoater 2 machine to obtain a Parylene-C thickness of 3 μm. Substrates were then patterned with a 20 μm layer of AZ 9260 photoresist and AZ developer (AZ Electronic Materials). Etching was performed using RIE Oxford 80 Plasmalab Plus with a mixture O_2_ and CHF_3_ (10:1, 160 W, 30 mTorr) for Parylene-C and O_2_ and SF_6_ (1:4, 300 W, 70 mTorr) for PDMS. The PEDOT:PSS mixture was spin-coated accordingly to achieve a thickness of 300 nm. Devices were subsequently baked for 1 hour at 130 °C.

### Mechanical/electrical characterisation

Stretching tests were performed on a custom-designed XY motion table with motors from ETEL as shown in Fig. SI-3. The device under test was clamped between two 3D-printed plastic jaws. The speed of the motor was set at 100 µm/s. Electrical measurements of the resistance of the lines were performed using a four-point probe setup using pogo pins, while for the PEDOT:PSS formulation optimisation characterisation, the measurement was performed using copper strips instead of pogo pins. The distance between the jaws were 40 mm. The setup was connected to a Keithley® 2636 A source measure unit. IV curves of the OECT were performed using an external Ag/AgCl gate immersed in a 0.01 M KH_2_PO_4_ solution, applying a voltage through the Keithley® and collecting the voltage between the source and drain. The transistor channel length is 250 µm, the contact lines are 400 µm wide with a PEDOT:PSS thickness of 300 µm. Electrical measurements, displacement, and data collection were performed simultaneously using a homemade LabVIEW script.

### Data availability

All data generated and analysed during this study are either included in the published article itself (or available within the Supplementary Information files).

## Electronic supplementary material

SI_Figure 1

Supplementary Information

## References

[1] Sun Y, Rogers JA (2007). Inorganic Semiconductors for Flexible Electronics. Adv. Mater..

[2] Hsu PI (2002). Thin-film transistor circuits on large-area spherical surfaces. Appl. Phys. Lett..

[3] Lu N, Kim D-H (2014). Flexible and Stretchable Electronics Paving the Way for Soft Robotics. Soft Robot..

[4] Sekitani T (2008). A Rubberlike Stretchable Active Matrix Using Elastic Conductors. Science.

[5] Kim J (2014). Stretchable silicon nanoribbon electronics for skin prosthesis. Nat. Commun..

[6] *Stretchable Bioelectronics for Medical Devices and Systems*. (Springer International Publishing, 2016).

[7] Ahn J-H, Je JH (2012). Stretchable electronics: materials, architectures and integrations. J. Phys. Appl. Phys..

[8] Johnston ID, McCluskey DK, Tan CKL, Tracey MC (2014). Mechanical characterization of bulk Sylgard 184 for microfluidics and microengineering. J. Micromechanics Microengineering.

[9] Hocheng H, Chen C-M (2014). Design, Fabrication and Failure Analysis of Stretchable Electrical Routings. Sensors.

[10] Kim Y (2013). Stretchable nanoparticle conductors with self-organized conductive pathways. Nature.

[11] Matsuhisa N (2015). Printable elastic conductors with a high conductivity for electronic textile applications. Nat. Commun..

[12] Lacour SP, Wagner S, Huang Z, Suo Z (2003). Stretchable gold conductors on elastomeric substrates. Appl. Phys. Lett..

[13] Wagner S (2004). Electronic skin: architecture and components. Phys. E Low-Dimens. Syst. Nanostructures.

[14] Watanabe M, Shirai H, Hirai T (2002). Wrinkled polypyrrole electrode for electroactive polymer actuators. J. Appl. Phys..

[15] Zhang Y (2014). Experimental and Theoretical Studies of Serpentine Microstructures Bonded To Prestrained Elastomers for Stretchable Electronics. Adv. Funct. Mater..

[16] Hirsch A, Michaud HO, Gerratt AP, de Mulatier S, Lacour SP (2016). Intrinsically Stretchable Biphasic (Solid-Liquid) Thin Metal Films. Adv. Mater..

[17] Gray DS, Tien J, Chen CS (2004). High-Conductivity Elastomeric Electronics. Adv. Mater..

[18] Brosteaux D, Axisa F, Gonzalez M, Vanfleteren J (2007). Design and Fabrication of Elastic Interconnections for Stretchable Electronic Circuits. IEEE Electron Device Lett..

[19] Sun Y, Choi WM, Jiang H, Huang YY, Rogers JA (2006). Controlled buckling of semiconductor nanoribbons for stretchable electronics. Nat. Nanotechnol..

[20] Kim D-H (2008). Materials and noncoplanar mesh designs for integrated circuits with linear elastic responses to extreme mechanical deformations. Proc. Natl. Acad. Sci..

[21] Lu N, Yang S (2015). Mechanics for stretchable sensors. Curr. Opin. Solid State Mater. Sci..

[22] Jahanshahi A (2013). Stretchable Circuits with Horseshoe Shaped Conductors Embedded in Elastic Polymers. Jpn. J. Appl. Phys..

[23] Axisa, F., Bossuyt, F., Vervust, T. & Vanfleteren, J. Laser based fast prototyping methodology of producing stretchable and conformable electronic systems. In *Electronics System-Integration Technology Conference, 2008. ESTC 2008*. 2nd 1387–1390 (IEEE, 2008).

[24] Yang S, Ng E, Lu N (2015). Indium Tin Oxide (ITO) serpentine ribbons on soft substrates stretched beyond 100%. Extreme Mech. Lett..

[25] Hsu Y-Y (2011). The effects of encapsulation on deformation behavior and failure mechanisms of stretchable interconnects. Thin Solid Films.

[26] Li T, Suo Z, Lacour SP, Wagner S (2005). Compliant thin film patterns of stiff materials as platforms for stretchable electronics. J. Mater. Res..

[27] Macaya DJ (2007). Simple glucose sensors with micromolar sensitivity based on organic electrochemical transistors. Sens. Actuators B Chem..

[28] Ramuz M (2014). Combined Optical and Electronic Sensing of Epithelial Cells Using Planar Organic Transistors. Adv. Mater..

[29] Ramuz M, Hama A, Rivnay J, Leleux P, Owens RM (2015). Monitoring of cell layer coverage and differentiation with the organic electrochemical transistor. J Mater Chem B.

[30] Jimison LH (2012). Measurement of Barrier Tissue Integrity with an Organic Electrochemical Transistor. Adv. Mater..

[31] Stavrinidou E, Sessolo M, Winther-Jensen B, Sanaur S, Malliaras GG (2014). A physical interpretation of impedance at conducting polymer/electrolyte junctions. AIP Adv..

[32] Kergoat L, Piro B, Berggren M, Horowitz G, Pham M-C (2012). Advances in organic transistor-based biosensors: from organic electrochemical transistors to electrolyte-gated organic field-effect transistors. Anal. Bioanal. Chem..

[33] Tarabella G (2010). Effect of the gate electrode on the response of organic electrochemical transistors. Appl. Phys. Lett..

[34] Khodagholy, D. *et al*. High transconductance organic electrochemical transistors. *Nat. Commun*. **4** (2013). 10.1038/ncomms3133 PMC3717497 23851620

[35] Sessolo M (2013). Easy-to-Fabricate Conducting Polymer Microelectrode Arrays. Adv. Mater..

[36] Strakosas X, Bongo M, Owens RM (2015). The organic electrochemical transistor for biological applications. J. Appl. Polym. Sci..

[37] Chen W, Lam RHW, Fu J (2012). Photolithographic surface micromachining of polydimethylsiloxane (PDMS). Lab Chip.

[38] Moon GD (2013). Highly Stretchable Patterned Gold Electrodes Made of Au Nanosheets. Adv. Mater..

[39] Hyun WJ (2015). Screen Printing of Highly Loaded Silver Inks on Plastic Substrates Using Silicon Stencils. ACS Appl. Mater. Interfaces.

[40] Jiang J (2016). Fabrication of Transparent Multilayer Circuits by Inkjet Printing. Adv. Mater..

[41] Kim T-H (2009). Kinetically controlled, adhesiveless transfer printing using microstructured stamps. Appl. Phys. Lett..

[42] Kim JT (2012). Three-Dimensional Writing of Highly Stretchable Organic Nanowires. ACS Macro Lett..

[43] Zhang S (2017). Patterning of Stretchable Organic Electrochemical Transistors. Chem. Mater..

[44] Lipomi DJ, Tee BC-K, Vosgueritchian M, Bao Z (2011). Stretchable Organic Solar Cells. Adv. Mater..

[45] Lapczyna M, Chen KP, Herman PR, Tan HW, Marjoribanks RS (1999). Ultra high repetition rate (133 MHz) laser ablation of aluminum with 1.2-ps pulses. Appl. Phys. A.

[46] Gonzalez M (2008). Design of metal interconnects for stretchable electronic circuits. Microelectron. Reliab..

[47] DeFranco JA, Schmidt BS, Lipson M, Malliaras GG (2006). Photolithographic patterning of organic electronic materials. Org. Electron..

[48] Anenden, M. P., Svehla, M., Lovell, N. H. & Suaning, G. J. Process development for dry etching polydimethylsiloxane for neural electrodes. In *Engineering in Medicine and Biology Society, EMBC, 2011 Annual International Conference of the IEEE* 2977–2980 (IEEE, 2011). 10.1109/IEMBS.2011.6090817 22254966

[49] Vlachopoulou M-E, Kokkoris G, Cardinaud C, Gogolides E, Tserepi A (2013). Plasma Etching of Poly(dimethylsiloxane): Roughness Formation, Mechanism, Control, and Application in the Fabrication of Microfluidic Structures. Plasma Process. Polym..

[50] Vosgueritchian M, Lipomi DJ, Bao Z (2012). Highly Conductive and Transparent PEDOT:PSS Films with a Fluorosurfactant for Stretchable and Flexible Transparent Electrodes. Adv. Funct. Mater..

[51] Lipomi DJ (2012). Electronic Properties of Transparent Conductive Films of PEDOT:PSS on Stretchable Substrates. Chem. Mater..

[52] Chen C (2011). Mechanical characterizations of cast Poly(3,4-ethylenedioxythiophene):Poly(styrenesulfonate)/Polyvinyl Alcohol thin films. Synth. Met..

[53] ElMahmoudy M (2017). Tailoring the Electrochemical and Mechanical Properties of PEDOT:PSS Films for Bioelectronics. Macromol. Mater. Eng..

[54] Provided by Clevios PH1000 maker, Haraeus. Available at: http://www.clevios.com/index.php?page_id=3671.

