# Supplementary material for: Laser-patterned metallic interconnections for all stretchable organic electrochemical transistors
Source: Sci Rep. 2018 May 31;8:8477. doi: 10.1038/s41598-018-26731-8 (PMC5981432; doi:10.1038/s41598-018-26731-8)
Supplement: Supplementary file 2 — Supplementary Information [file 41598_2018_26731_MOESM2_ESM.docx]

# Supplementary information

**Laser-patterned metallic interconnections for all stretchable organic electrochemical transistors**

*Bastien Marchiori, Roger Delattre, Stuart Hannah, Sylvain Blayac, Marc Ramuz**

*See attached video file*

**SI, Figure 1**.Video of the process used to perform the laser cutting. The design is loaded into the software and then the laser is applied 3 times to completely cut the aluminium layer. This process avoids the use of photolithography, and is ultimately faster and cheaper.

a

b

**SI, Figure 2**. Optimisation of the formulation for the PEDOT:PSS layer, spin-coated on a PDMS substrate. a) Resistance as a function of the strain for different wt% of PVA. b) Resistance as a function of the strain for different vol% of Capstone®.


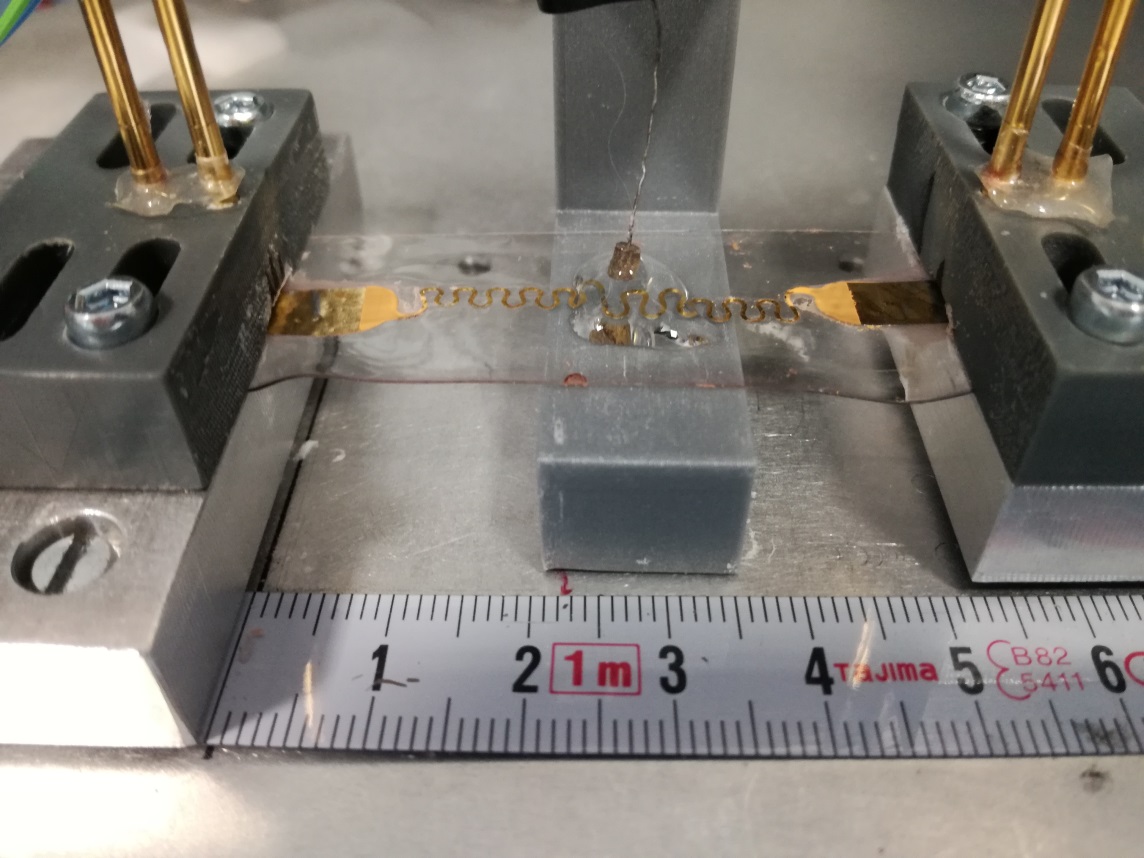


**SI, Figure 3**. Photograph of the setup used to characterise the OECT. The device is clamped at either end. One output from the Keithley® is connected to the source and the drain. A second output is connected to an Ag/AgCl electrode immersed in an electrolyte. The strain and the electrical measurements are synchronised with LabVIEW.

29%

38%

19%

0%

11%

40%

**SI, Figure 4**. Output curves of the best performing device for different strains up to 40 %, where the OECT no longer shows transistor behaviour. The current drops by a factor 10 between 0 % and 38 % strain.

**SI, Figure 5.** Transconductance as a function of V_GS_ associated with the output curves in SI, Figure 4. The maximum transconductance can reasonably be associated to the point between V_GS_ = 0V and V_GS_ = 0.05V.

11%

0%

35%

19%

42%

**SI, Figure 6**. Output curves for a device featuring a channel length of 250 µm and a 500 nm-thick layer of PEDOT:PSS. The device shows good performance until 19 % strain and then suddenly drops. This is possibly due to a reorganisation of the polymer chains. The first measurement with V_GS_ = 0 V is completely different from the other datasets, resulting in negative transconductance in some cases. The other curves are still showing transistor behaviour. The transconductance plot has been extracted from V_GS_ = 0 V and V_GS_= 0.5 V.
